# Draft Genome Sequence of *Bacillus tequilensis* Strain FJAT-14262a

**DOI:** 10.1128/genomeA.01317-15

**Published:** 2015-11-12

**Authors:** Qian-Qian Chen, Bo Liu, Guo-hong Liu, Jie-ping Wang, Jian-Mei Che

**Affiliations:** Agricultural Bio-Resources Research Institute, Fujian Academy of Agricultural Sciences, Fuzhou, Fujian, China

## Abstract

*Bacillus tequilensis* FJAT-14262a is a Gram-positive rod-shaped bacterium. Here, we report the 4,038,551-bp genome sequence of *B. tequilensis* FJAT-14262a, which will provide useful information for genomic taxonomy and phylogenomics of *Bacillus*.

## GENOME ANNOUNCEMENT

The genus *Bacillus* is the most diverse and prominent genus of aerobic endospore-forming bacteria (1), consisting of ~299 species and 7 subspecies until 2015 (List of Prokaryotic names with Standing in Nomenclature. http://www.bacterio.net/bacillus.html). Some *Bacillus* components have important roles in industrial processes, such as the transglutaminase of *Bacillus subtilis* and biofilm formation of butyric acid (2, 3). *Bacillus* strains are widely distributed in the environment. Strain FJAT-14262a was isolated from rhizosphere soil of *Anoectochilus roxburghii* in China and identified as *Bacillus tequilensis*, which is a phylogenetically close neighbor of *B. subtilis* according to their 16S rRNA gene sequence similarity. The best growth of *B. tequilensis* FJAT-14262a was achieved at 35°C and pH 8. Here, we present the high-quality draft genome sequence of *B. tequilensis* FJAT-14262a.

The genome sequencing of *B. tequilensis* FJAT-14262a was performed via the Illumina HiSeq 2500 system. Two DNA libraries with insert sizes of 500 and 5,000 bp were constructed and sequenced. The genome coverage was approximately 150-fold coverage. The reads were assembled via the SOAP*denovo* software version 2.10 (4), using a key parameter K setting at 77. Through the data assembly, 7 scaffolds with a total length of 3,853,296 bp were obtained, and the contig *N*_50_ was 185,225 bp. The average length of the scaffolds was 550,470 bp, and the longest and shortest scaffolds were 1,047,314 bp and 711 bp, respectively. A total 79.77% clean reads were aligned back to the genome, which covered 99.57% of the sequence.

Annotation of the genome was performed using the NCBI Prokaryotic Genome Annotation Pipeline (PGAP) (http://www.ncbi.nlm.nih.gov/genomes/static/Pipeline.html) utilizing the GeneMark, Glimmer, and tRNAscan-SE tools (5). A total of 4,029 genes were predicted, including 3,919 coding sequences (CDSs), 41 pseudogenes, 66 tRNAs, 6 rRNAs, and 1 noncoding RNA (ncRNA) gene. There were 3,090 genes assigned to the COG database. The average DNA G+C content was 43.71%.

### Nucleotide sequence accession numbers.

This whole-genome shotgun project has been deposited at DDBJ/EMBL/GenBank under the accession no. LGRW00000000. The version described in this paper is version LGRW00000000.1.
